# Increasing the (Re)Activity of MgO for Carbon Capture under Continuous Ball Milling

**DOI:** 10.1021/acs.chemmater.6c01597

**Published:** 2026-10-08

**Authors:** Emanuele Antico, Thibaud Aumond, Marc Meyer, Linfeng Li, Eko Budiyanto, Ferdi Schüth

**Affiliations:** Max-Planck-Institut für Kohlenforschung, Kaiser-Wilhelm-Platz 1, 45470, Mülheim an der Ruhr, Germany

## Abstract

Carbon capture and storage from point emission sources is a crucial technology able to mitigate the progress of climate change by slowing down the emission of greenhouse gases. Current technologies, such as amine scrubbing and calcium looping, face challenges including high energy consumption and limited efficiency under humid conditions. Magnesium oxide (MgO) is a promising CO_2_ sorbent due to its lower decarboxylation enthalpy compared to calcium oxide, but its practical use is hindered by slow CO_2_ uptake kinetics and the formation of a passivating carbonate layer that inhibits further CO_2_ diffusion. While synthetic methods have improved MgO surface area, scalability remains an issue. This study addresses the kinetic limitations of MgO by investigating how continuous ball milling can reduce the performance hindrance from the passivating-carbonate layer on MgO and enhance CO_2_ uptake under industrially relevant conditions. Here we show that continuous ball milling of MgO under a CO_2_ atmosphere significantly increases its CO_2_ uptake capacity. Unlike conventional approaches that rely on presynthesized high-surface-area MgO, our results demonstrate that ball milling during the sorption step does not only increase the specific surface area of MgO but also actively enhances its CO_2_ capture performance, with a 6-fold increase in adsorption capacity compared to static exposure. The addition of water further boosts performance, doubling uptake under simulated flue gas conditions (10% CO_2_, 40 °C), likely due to the formation of bicarbonates and hydroxycarbonates. The performance stability of MgO under continuous milling and moisture conditions was evaluated in several sorption-regeneration cycles, showing consistent CO_2_ uptake over ten cycles with an average sorption capacity of 17.4 wt %. Structural analyses reveal that carbonation proceeds via magnesium hydroxycarbonate species, which fully decompose upon regeneration, preserving sorbent stability over ten cycles. This study underscores the practical relevance of mechanochemical activation for enhancing the capture capacity of MgO, offering a promising approach for industrial point-source CO_2_ capture applications.

## Graphical abstract

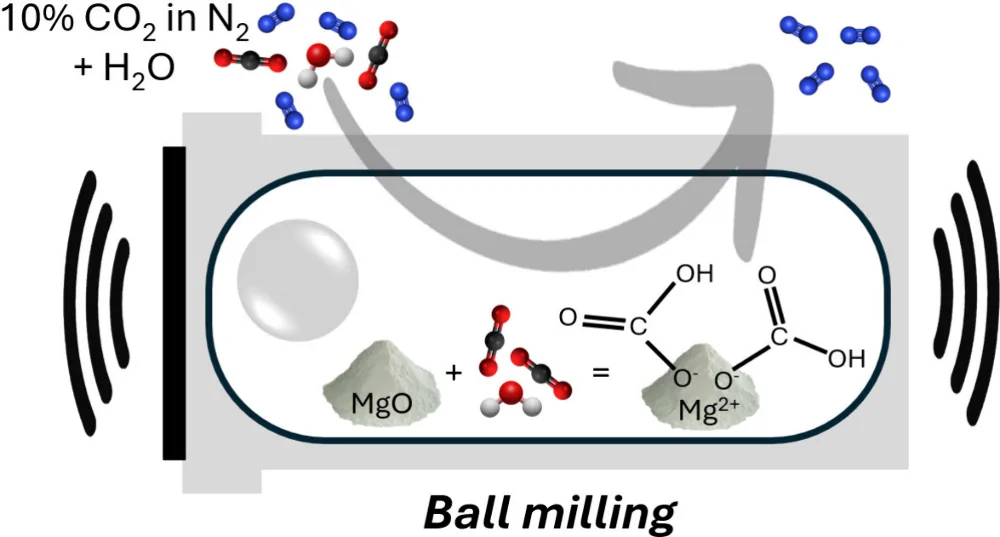

## Introduction

The reduction of CO_2_ emissions is a crucial aspect in mitigating the influence of climate change on the planet and the environment. In addition to avoiding CO_2_ emissions altogether, CO_2_ capture and storage or use (CCS/CCU) is a valuable option.^1^ While direct air capture seems, in the near term, both thermodynamically and economically unattractive, mitigating emissions from point sources in hard-to-abate sectors could play a relevant role in swiftly reducing CO_2_ emissions and decelerating the pace of climate change.^2^

Today, numerous mature technologies are already being implemented industrially where economically feasible, such as amine scrubbing^3–6^ and calcium looping.^7–10^ These technologies have been proven compatible with the hot, moist, and complex flue streams from steel factories and coal-fired power plants at various scales. Indeed, depending on the factories, different gas mixtures can be encountered, with different CO_2_ concentrations, moisture levels, and impurities. High-concentration capture methods include precombustion capture and oxy-fuel combustion capture with CO_2_ concentration between 15–50%. Postcombustion capture, on the other hand, deals with lower CO_2_ concentrations, usually between 2 and 15%. The capture of CO_2_ from flue gases must deal with the presence of humidity that can reduce adsorption capacities.^11^ Another problem that still is burdening the implementation of carbon capture technologies besides fluctuating spot CO_2_ price is the relatively energy-intensive regeneration of the sorbents, which diminishes the overall CO_2_ capture efficiency unless the energy utilized is fully renewable. Research is therefore embracing the challenge to develop more energy-efficient and innovative technologies for carbon capture, such as, for example, using Metal–Organic-Frameworks (MOFs) as sorbents, which hold promise, but are currently facing the challenge of reaching cost-effective implementation, and sharp reduction of adsorption due to the presence of water.^12–14^

A significant alternative that is worth developing is switching from calcium to magnesium looping, making use of MgO as a solid sorbent. In fact, the enthalpy of decarboxylation of MgCO_3_ is only one-third of that of CaCO_3_.^15^ However, despite these potential advantages, the utilization of MgO as a carbon capture agent presents significant challenges that hinder its implementation: it has unfavorable CO_2_ sorption kinetics that make the CO_2_ uptake comparatively slow. This is due to the well-known quick carbonation of the surface basic sites of MgO, forming an outer layer of MgCO_3_ in the presence of CO_2_, which prevents further diffusion of CO_2_ into the MgO core. This is, in fact, in agreement with the observation that the CO_2_ capture performance of MgO is strongly linked to its surface area.^16^

This is also consistent with findings of many studies, observed already since the 1970s, that MgO materials offer a CO_2_ sorption capacity of approximately 10 mg g^–1^ when using commercial MgO.^17^ The theoretical capacity, on the other hand, is much higher with 1090 mg g^–1^ for the formation of MgCO_3_. To mitigate this low uptake issue due to carbonate formation, many research groups are investigating synthetic pathways for increasing the surface area of MgO in order to reduce the fraction of the poorly accessible core of MgO particles. Contributions in this direction focus on new synthetic methods for the synthesis of porous MgO (leading to capture results in the range around 30–200 mg_CO2_ g_MgO_^–1^, using pure CO_2_ gas flow) or on supporting MgO on other materials.^18–26^ While significant improvements have been achieved, these methods often conflict with scalability, because such syntheses are not well-suited for large-scale industrial applications, where commercially available bulk amounts of MgO would be required. We hypothesize that breaking the core–shell structure formed after initial carbonation could ’refresh’ the MgO surface area exposed to the CO_2_ atmosphere, thereby overcoming the aforementioned kinetic bottleneck.

Therefore, we decided to investigate whether the CO_2_ uptake of MgO could be improved via ball milling, which could potentially be well-suited to address these problems. This electricity-based technology is expected to have an almost zero CO_2_ footprint in the future, driven by the growing adoption of low-carbon energy sources (e.g., solar, wind, and hydroelectric power), as mandated by institutional policies. This shift will eventually enable a more sustainable process. Furthermore, for low-concentration carbon capture technologies, such as direct air capture, one could run CO_2_ sorption plants only at times when there is an electricity surplus. As of today, research is trying to leverage mechanochemical methods in carbon capture working on the synthesis of materials for CO_2_ capture^27,28^ or of performance improvement of CO_2_ capturing materials with positive results. For example, in 2016, the group of Perez-Ramirez presented a scalable route based on wet mechanochemical activation to synthesize highly effective MgO-stabilized (up to 15 wt %) CaO-based CO_2_ sorbents that exhibited superior uptake capacity over numerous cycles.^29^ Valverde et al. also showed that milling CaO has a significant effect on its crystallite size and morphology, influencing its carbon capture performance under calcium looping conditions, strongly depending on the type of milling used.^30^ Beyond mechanochemical synthesis of materials for capture, in situ ball milling could be used for the capture process itself. The groups of Lim, Li, and Baek showed that using commercial zirconium oxide (ZrO_2_) and nickel catalysts, the mechanochemical CO_2_ capture capacity was 75-fold higher than the conventional thermochemical process,^31^ reaching slightly above 100 mg g^–1^.

In this study, first, the impact of specific surface area on capture performance was studied by varying the mesh size of MgO with or without milling. After optimizing milling conditions and to access the sorption capacities of MgO under realistic industrial applications, we finally investigated the use of mechanochemistry for CO_2_ capture by mimicking flue gas conditions over several cycles and dealing with the presence of water.

## Methods

MgO of different sizes from Sigma-Aldrich was calcined at 600 °C for 6 h and stored in the glovebox prior to use. Pure CO_2_ (AirLiquide, 99.995%) and N_2_ (AirLiquide 99.999%) were used as received. The milling apparatus used in the experiments was a Retsch MM400, and the stainless steel jars were manufactured in house.^32^ Flow experiments were performed in an in-house built setup previously described.^33^

All the experiments were conducted in the stainless-steel milling jar dried overnight in an oven at 60 °C and then transferred into a glovebox. Calcined MgO was loaded into the jar together with the milling balls. A PTFE sealing ring was installed, and the jar was sealed by hand. In screening experiments to find the best milling conditions, two types of experiments were conducted. On one hand, for control experiments using premilled MgO subsequently exposed to CO_2_, a sealed milling jar containing 1.0 g of MgO and stainless-steel (SS) balls of 10 mm was placed in the mixer mill and milled at 20 Hz for 1 h using an MM400 milling machine. After milling, a preprepared balloon containing 2–3 g of CO_2_ was connected to the jar. The system was then kept under static conditions (no milling) for 24 h. On the other hand, for experiments performed under CO_2_ during milling, the sealed jar containing 1.0 g of MgO was first removed from the glovebox and a preprepared CO_2_ balloon was connected. The jar was then mounted in the milling machine, and milling was carried out at 20 Hz for 1 h. To verify the reliability of the method, certain experiments were performed twice (error bars in the graphs).

To further demonstrate the relevance of this approach for industrial point-source applications, experiments using a flow system mimicking flue gas were performed. The setup used was described elsewhere,^33^ and the best conditions found previously were employed (MgO mesh size, frequency, type of ball). Briefly, a gas cylinder containing 10% CO_2_ in N_2_ was connected to the milling jar containing the balls and 0.8 g of MgO. Two bubblers filled with Raschig rings and distilled water were heated at 40 °C to generate a desired amount of water in the flow. Water content was calculated from the equilibrium vapor pressure of water at the corresponding temperature. Flow rate was set to 30 mL min^–1^ to avoid pressure drop and maximize the amount of CO_2_ and water sent to the jar. The milling was performed for 4 h. A thermocouple was placed inside of the jar to check the temperature, increasing from 30 °C at the beginning of the experiment to 40 °C at the end of the experiment. This temperature does not account for the generation of potential “hot spots” during collisions between the balls, the jar wall, and the materials, where, for a range of different materials, a maximum temperature of 200 °C for a few milliseconds at the impact point has been measured using a fast IR-camera.^34^ However, this temperature is insufficient to desorb CO_2_.

To study the recycling adsorption performances of MgO and its robustness after several cycles, the leftover MgO was calcined at 600 °C under argon and reused for subsequent cycles. In order to keep the same ball to powder weight ratio (BPR) which can influence the properties of MgO during milling, a small amount of fresh MgO to replace the amount needed for analysis was added to the calcined one after each cycle.

At the end of every milling, the jar was opened, the resulting solid was recovered, and a sample of it was used for quantitative analysis. A known amount of material resulting from the milling (50–100 mg) was weighed and transferred to a 2-necked round-bottom flask equipped with a septum and a stir bar. The round-bottom flask was closed with a cap, cooled down in an ice bath for 15 min, and connected through silicone tubing to a gas buret. The stirring was started, and 2 mL of a preprepared 6 M H_2_SO_4_ solution was added dropwise to the material, which led to CO_2_ evolution. Before the experiment, the gas buret was set to 150 mL and the Hg-piston was pushed toward smaller values by the evolving gas. After gas evolution terminated, the resulting volume of CO_2_ evolved would result from (1):

1 $$V_{{CO}_{2}}=150ml-V_{reached}-2ml$$

where V_reached_ is the volume indicated by the Hg-piston. The V_CO2_ was then converted to mass using the approximation of ideal gases, using the molar volume of CO_2_ at 20 °C, which is 24056 mL mol^–1^, and molar mass (2):

2 $$m_{{CO}_{2}}=V_{{CO}_{2}}÷24056mL,{mol}^{-1}\times {MW}_{{CO}_{2}}$$

The powders after capture were analyzed by Fourier transform infrared (FTIR) spectroscopy using a PerkinElmer-Spectrum Two (PerkinElmer, Waltham, USA) spectrometer equipped with an attenuated total reflectance (ATR) accessory. Samples were directly measured on the surface of the ATR crystal (diamond) at room temperature, recording 16 scans per spectrum with a resolution of 4 cm^–1^ in the range of 650–4000 cm^–1^. Samples were placed in an oven at 100 °C for 48 h before measurement to remove physisorbed water.

All samples for powder X-ray diffraction (PXRD) analysis were prepared by filling 0.5 mm borosilicate glass capillaries (WJM-Glas Müller GmbH). PXRD analyses were performed on a STOE StadiP transmission diffractometer using Cu radiation (1.5406 Å). The instrument is equipped with a primary Ge (111) monochromator (CuKα1) and a position-sensitive Mythen1K detector. Data were collected in the range between 5 and 80° (2θ) with a step width of 0.4° (2θ).

N_2_ physisorption at 77 K was performed using a Micromeritics 3Flex Surface Characterization Analyzer. Prior to analysis, samples were degassed at 200 °C overnight. The specific surface area was calculated from the adsorption data in the relative pressure range of 0.05 to 0.3 using the Brunauer–Emmett–Teller (BET) method.

Transmission electron microscopy (TEM) images were captured using a Thermo Scientific Talos F200X (S)­TEM microscope, with an acceleration voltage of 200 kV and a high-brightness field-emission gun (X-FEG). EDX analysis was recorded with a windowless Super-X EDX system equipped with 4 silicon drift detectors (SDDs).

Thermogravimetric analysis coupled with mass spectrometry (TGA-MS) analyses were performed using a STA 449 F1 Jupiter apparatus (Netzsch) to follow the mass loss. Under an argon flow of 20 mL min^–1^, the sample was heated up to 1000 °C with a rate of 3 °C min^–1^. Once the final temperature was reached, the plateau was held for 10 min. Released species, i.e., H_2_O (*m*/*z* = 18) and CO_2_ (*m*/*z* = 44), were monitored by mass spectra (QMS 403 D Aëolos, Netzsch).

## Results and Discussion

To initiate the investigation and test the hypothesis that milling may positively influence the CO_2_ uptake performance of MgO by increasing the specific surface area as reported by Gutiérrez-Bonilla and co-workers,^35^ three MgO of different particle sizes were milled with 10 mm SS balls at 20 Hz in a batch reactor for 1 h under argon. Independent of the initial particle size, an increase on the specific surface area was observed after milling (Figure 1a), with the highest value of 332 m^2^ g^–1^ obtained for the smallest particle size (325 mesh) (+140% in comparison with nonmilled one). CO_2_ sorption experiments using these three particle sizes were performed after and during milling. A substantially higher CO_2_ uptake was observed when milling commercial MgO for just 1 h under a CO_2_ atmosphere, in comparison with the material milled under an argon atmosphere followed by 24 h of static CO_2_ exposure under similar conditions (Figure 1b), and confirming the relevance of milling on sorption capacities. This was further confirmed in the experiment employing the smallest particle size of mesh 325, for which the adsorption capacity was 3 times higher when CO_2_ was adsorbed statically after 1 h of milling under inner gas, and more than six times higher when the CO_2_ was adsorbed during the milling. This demonstrated that the milling process is not simply increasing surface area but actively enhances CO_2_ capture. In addition to the fact that milling increased the surface area of MgO, a well-established effect for many types of different solids,^36–38^ the results showed that the amount of CO_2_ adsorbed was significantly higher when MgO was milled directly under a CO_2_ atmosphere. This also held when the total surface area was similar to samples milled under argon and then exposed to CO_2_ for a longer period of time.

**Figure 1:**
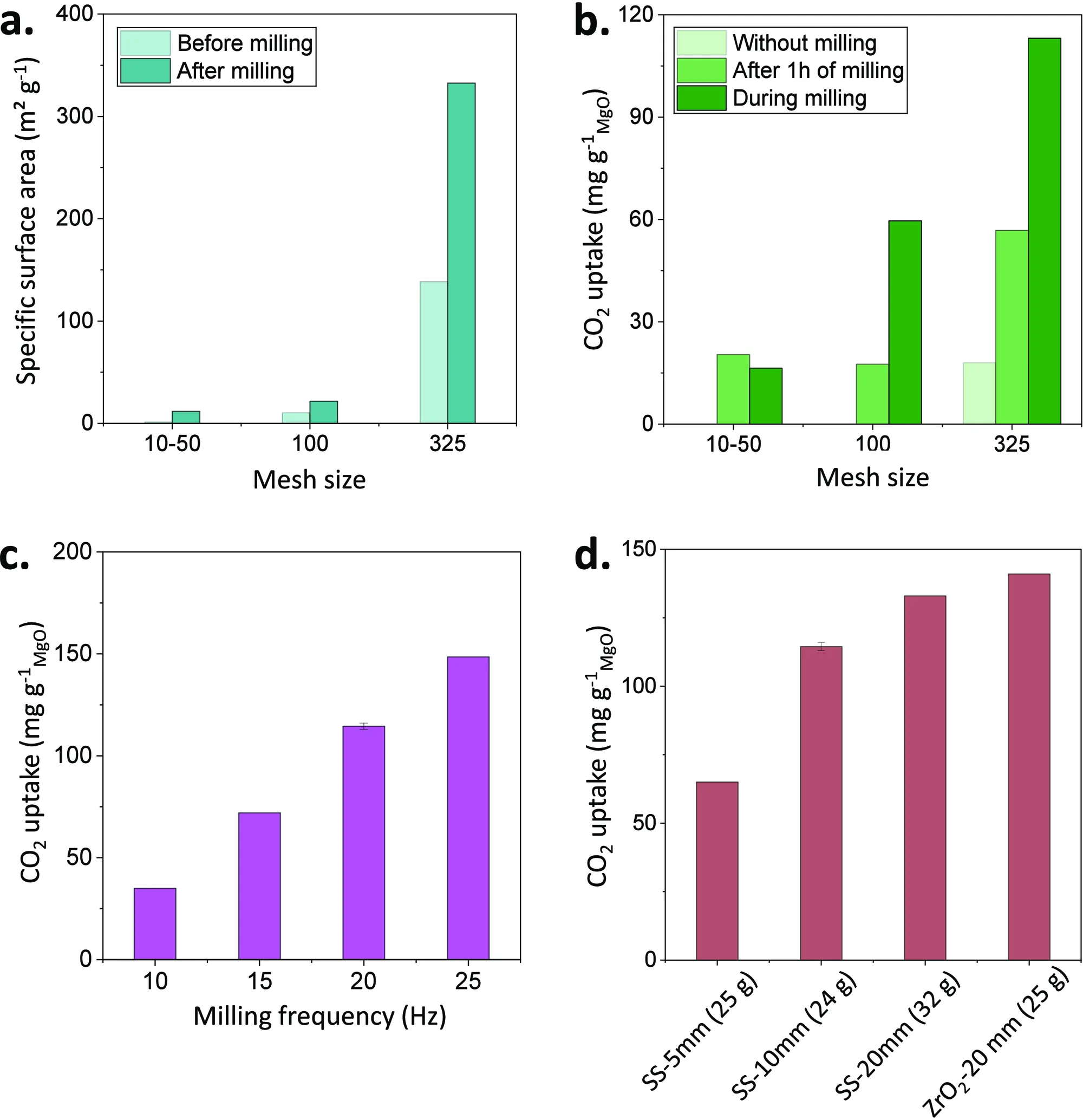
Screening capture conditions. a. Specific surface areas of different MgO before and after milling, b. CO_2_ capture performance of the different mesh sizes MgO without, after, and during milling using SS-10 mm balls and 20 Hz, c. CO_2_ capture performance for different milling frequency using 325 mesh size and SS-10 mm balls, and d. CO_2_ capture performance using different balls during milling at 20 Hz. SS = stainless steel.

Further investigations were continued with commercial MgO with a 325-mesh size to explore reaction parameters, including the impact of frequency or balls used. An increase in the frequency resulted in an increase of the amount of CO_2_ adsorbed, from 35 mg g^–1^ at 10 Hz to 148.5 mg g^–1^ at 25 Hz, using 10 mm SS balls (Figure 1c). However, the latter frequency led to significant abrasion of the stainless steel jar and balls, leading to a black powder (Figure S1). The highest frequency that did not significantly contaminate the sorbent material was 20 Hz, which was therefore selected for the rest of the study. Different ball sizes and compositions were investigated, and an increase in capture capacities was observed with ball diameter (Figure 1d). Indeed, balls smaller than 10 mm in diameter likely transfer kinetic energy less efficiently, while using a stainless-steel ball of 20 mm likely leads to a suboptimal MgO-to-milling material ratio. The use of a ZrO_2_ ball slightly increased the CO_2_ adsorption capacity (+6%), leading to a total of 141 mg g^–1^. For this reason, all of the following experiments were performed using a stainless-steel jar containing one ball of 20 mm ZrO_2_ and operated at a frequency of 20 Hz.

While the batch experiments allowed us to optimize milling conditions, the development of a flow system would be more relevant for practical applications, given the nature of postcombustion point source emission. To assess the potential of this approach under conditions closer to those encountered industrially, the milling jar has been connected to a flow setup, passing a gas mixture of 10% CO_2_ in N_2_. To mimic flue gas composition, water was added to the flow by using a two-bubblers-setup set at a temperature of 40 °C (Figure S2).

Previous studies showed that the presence of water could positively affect the CO_2_ capture process in the case of MgO as a sorbent. Indeed, since water can promote the formation of the bicarbonate ion instead of carbonate, its presence in the reaction environment could double the theoretical capture capacity from 1090 to 2180 mg g^–1^. Water-promoted capture by MgO-based materials has been previously studied by Bang et al., who showed that the OH groups resulting from hydration of the surface can enhance the adsorption of CO_2_.^39^

The use of water without milling enhanced CO_2_ sorption, leading to a total CO_2_ capture of 9.1 wt %, which is 2.5 times higher than without water (3.7 wt %). Furthermore, the use of milling led to higher CO_2_ capture, up to 17.5 wt %, which is almost 5 times higher than using CO_2_ without water and without milling (Figure 2a). By analyzing the resulting FT-IR spectra, an increase in intensity was observed for the bands localized at around 1430 and 1520 cm^–1^, which are attributed to the asymmetric in-plane stretching mode of the carbonate groups.^40^ The same observation was made for the band localized at around 850 cm^–1^, attributed to the out-of-plane bending mode of the carbonate groups.^41^ Nevertheless, while some bands were growing due to the increase in CO_2_ sorption, new bands appeared in the experiment using water, such as the shoulder at around 1640 cm^–1^ (Figure S3) and the wide band between 3000 and 4000 cm^–1^.

**Figure 2:**
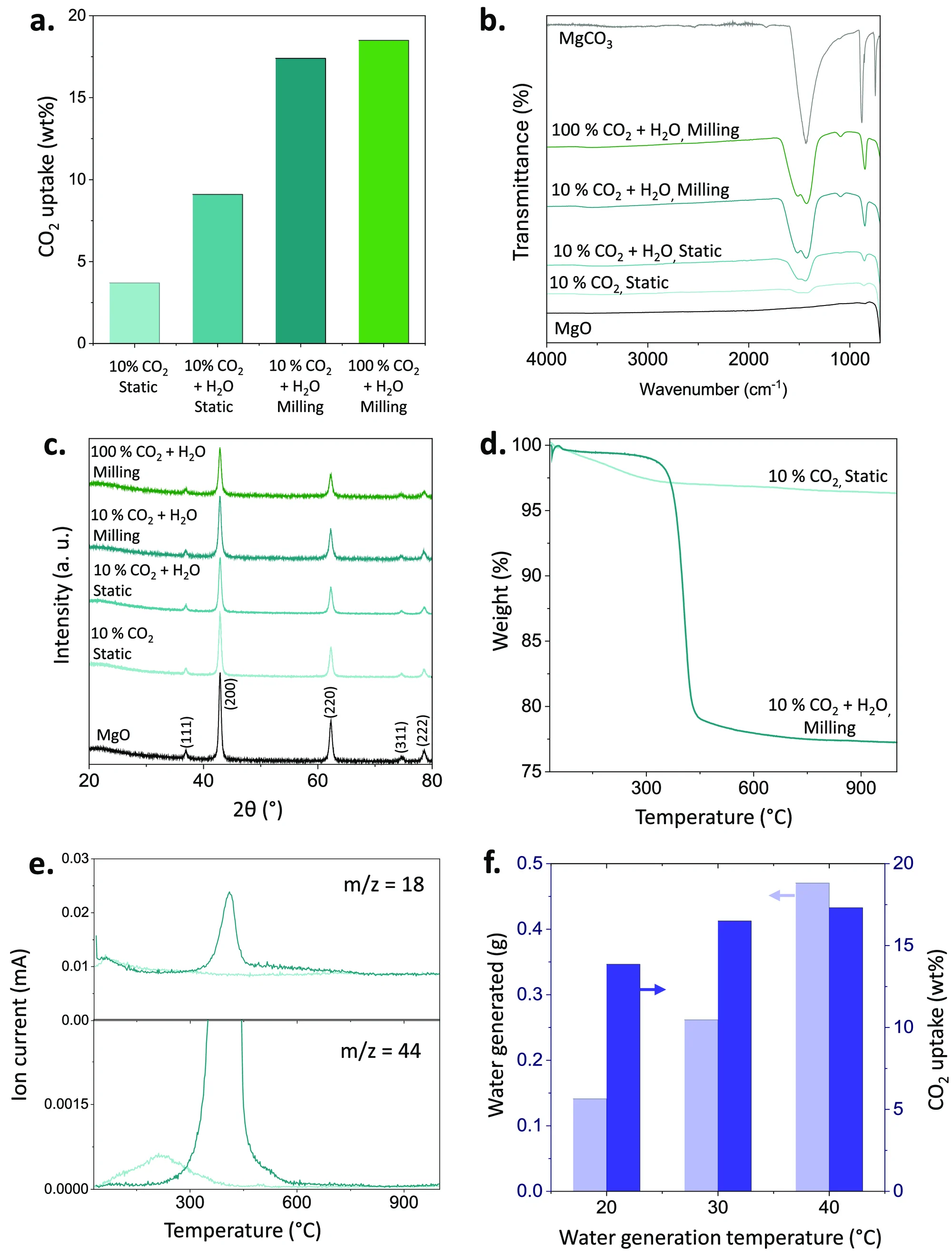
a. CO_2_ uptake on MgO using the flow setup with different conditions, b. resulting FT-IR spectra obtained from ATR analysis, and c. resulting XRD patterns. d. TGA profiles and e. resulting MS spectra of the sample exposed to 10% CO_2_ under static conditions (light turquoise) and the sample exposed to 10% CO_2_ passed through water and subjected to milling (dark turquoise). f. CO_2_ sorption capacity of MgO using different amount of water: light blue refers to H_2_O generated and dark blue to CO_2_ uptake.

These two bands correspond to the bending and stretching vibration modes of OH groups, respectively, and were attributed to the formation of bicarbonate species.^42^ However, a sharp band located near 3700 cm^–1^, attributed to the O–H stretching vibration of Mg­(OH)_2_, was expected, but not observed.^43,44^ Its absence indicates that Mg­(OH)_2_ reacts immediately with CO_2_ upon formation, as supported by the fact that the carbonation rate of Mg­(OH)_2_ indeed exceeds that of MgO.^45^ Moreover, it should be noted that only a small amount of water was passed through the milling jar during the experiment, corresponding to the saturated vapor pressure of water at 40 °C (Pv_sat_ = 7.38 kPa).

XRD experiments were performed on the three materials obtained after CO_2_ capture (Figure 2c), and they did not show any characteristic peaks of Mg­(OH)_2_ at around 2θ = 38 and 51°, confirming the absence of this phase.^46,47^ Only MgO-related peaks were observed. The XRD patterns of the milled samples showed no evidence of crystalline MgCO_3_ or other carbonate phases, implying the formation of amorphous magnesium hydroxycarbonates or carbonate species with crystallite sizes below the detection threshold.

TGA-MS was performed on the sample exposed to 10% CO_2_ under static conditions, as well as on the sample treated with water-containing 10% CO_2_ stream and subjected to milling (Figure 2d-e). In the first case, CO_2_ capture is confirmed by the presence of CO_2_ released between 40 and 400 °C in the TG experiment, with no water detected. In contrast, for sorbent used under milling in water-containing atmosphere, CO_2_ release shifts to higher temperatures and is accompanied by water in the released gas, confirming the formation of bicarbonate and/or hydroxycarbonate species.

Interestingly, the quantity of CO_2_ captured using 10% CO_2_ in N_2_ matched well with the one obtained using 100% CO_2_ gas flow without carrier gas, which led to the adsorption of 18.5 wt % CO_2_. FT-IR spectra and XRD patterns presented identical features, indicating that the sorption capacity of MgO was only little affected by CO_2_ concentration. On the other hand, both milling and moisture increased the adsorption capacity. To study the impact of water concentration, experiments were performed using different temperatures for water generation (Figure 2f). By reducing the temperature to 30 and 20 °C, a decrease in CO_2_ sorption was observed, confirming that the moisture concentration impacted CO_2_ sorption capacities.

Experiments were performed for different durations under the optimized conditions, and as expected, increasing the milling time under CO_2_ flow also increased CO_2_ capture, likely due to the formation of new sorption sites with water (Figure 3). However, this increase was not linear, and the kinetics of adsorption reduced with time. After 1 h of milling, the adsorption of CO_2_ reached 10 wt %, representing an increase of 10 wt % during the first hour. After 4 h of milling, the adsorption reached 17.4 wt %, whereas, after 8 h of milling, MgO could capture 21.3 wt % of CO_2_. The quantity adsorbed after 8 h corresponds to the adsorption of 295.3 mg of CO_2_ per gram of MgO, which is the highest value ever reported in the literature for commercial MgO.^18,48^ After 8 h, no fundamental differences were observed in the FT-IR spectrum in comparison with 4 h of milling, indicating that similar functional groups were detected, and only carbonate and bicarbonate species were formed.

**Figure 3:**
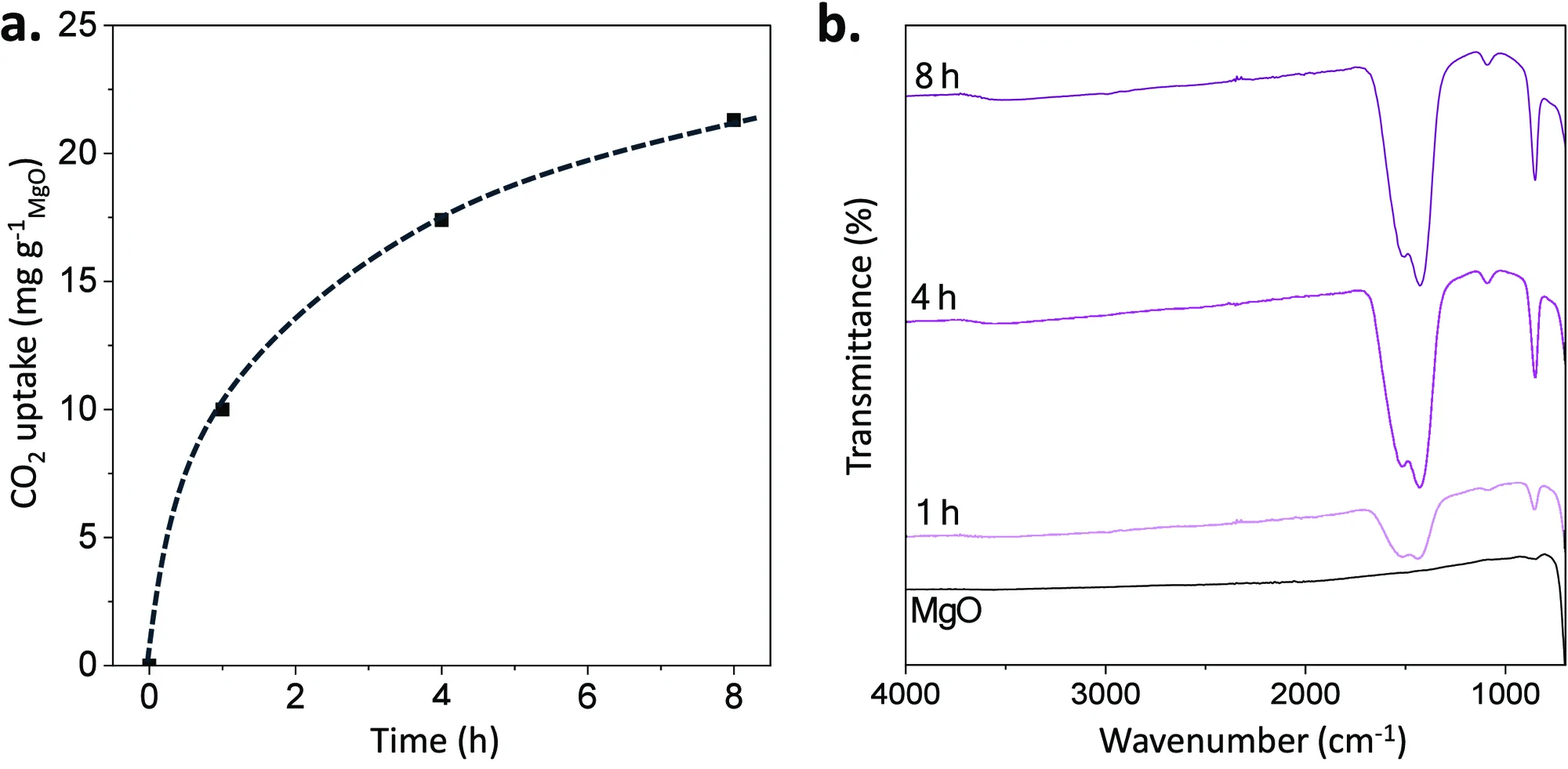
a. CO_2_ uptake as a function of time for flow experiments with 10% CO_2_ in N_2_ and water under milling. The dotted line is intended as a visual guide only and does not correspond to any fitting. b. FT-IR spectra of the resulting sorbent.

In any technical application, the sorbent needs to be recycled, and sorption capacity can be affected in cycling.^48,49^ For that reason, we checked the performance of MgO in repeated runs to determine whether the performance is stable or whether the sorbent would degrade. 0.8 g of MgO was milled with a 20 mm ZrO_2_ ball for 4 h in 10% CO_2_/N_2_ atmosphere using the bubbler setup for the generation of water, analyzed, regenerated, and reused for 10 rounds.

As shown in Figure 4a, the sorption performance of the MgO was nearly constant during these 10 cycles, with an average CO_2_ capture capacity of 17.4%. The XRD analyses performed after 1, 5, and 10 cycles exhibit no differences in the pattern of the sorbent, showing only the characteristic reflections of MgO, without amorphization (Figure 4b). The robustness of the sorbent after several cycles of milling was confirmed, even after each calcination step. While the material presented the characteristic bands of carbonate or bicarbonate after the milling using FT-IR spectroscopy, the calcined sample obtained after the last cycle did not present any of these bands, confirming the total regeneration of the sorbent after every cycle (Figure 4c). TEM image of MgO calcined at 600 °C without milling depicts an interconnected porous structure with a wall-thickness centered at around 19 nm (Figure 4d, Figure S4a). In contrast, the MgO regenerated after the first and last cycles possesses nanoparticle morphology with a broader particle size distribution centered at around 16 nm (Figure 4e-f, Figure S4b-c). The smaller particle size after ball-milling, as-observed from TEM images, is consistent with the increasing of specific surface area after ball-milling (Figure 1a). These TEM observations indicate that the mechanical force generated during ball-milling induces the morphological change of MgO.

**Figure 4:**
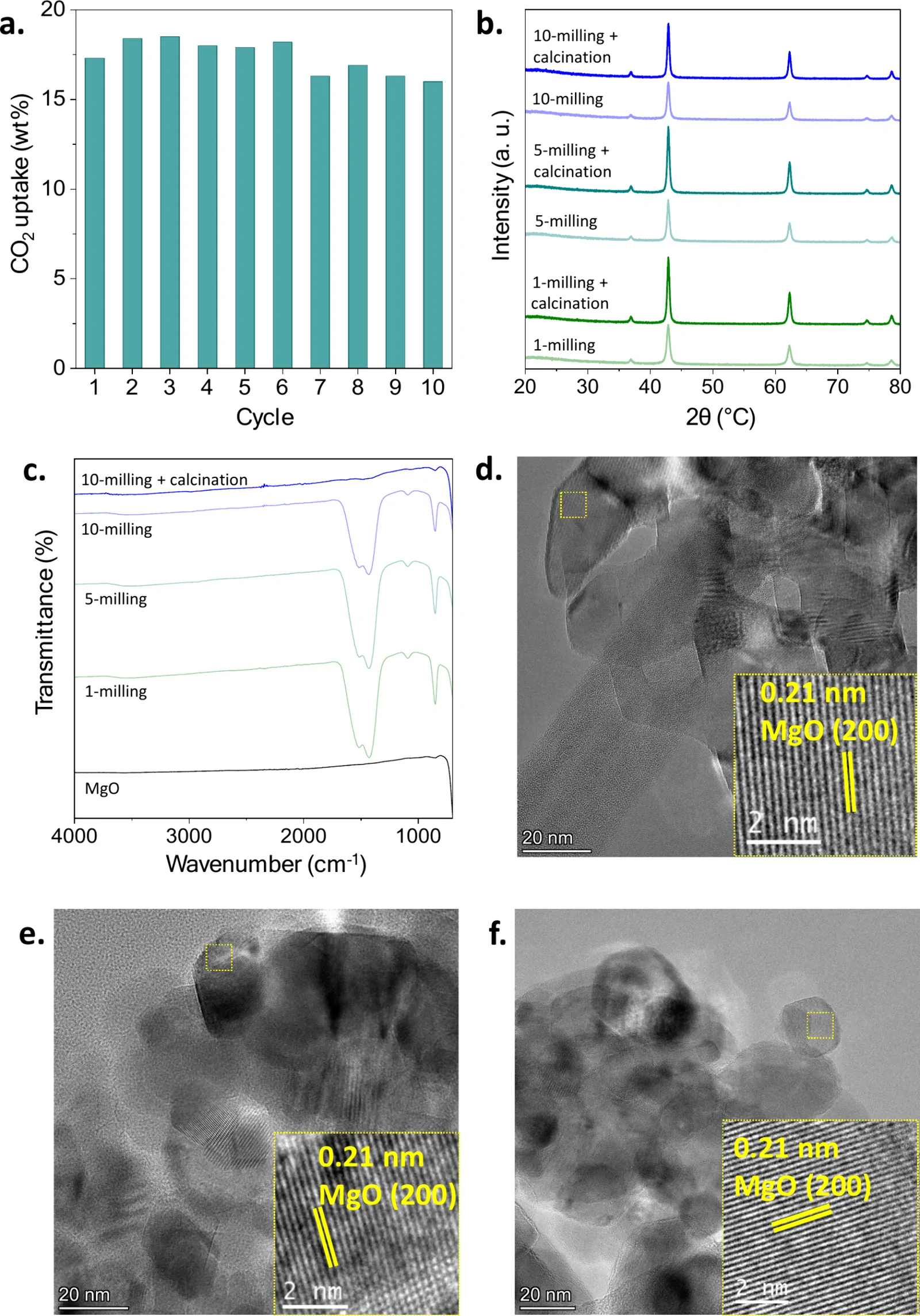
a. Cycling CO_2_ capture experiments with the wt % uptake, b. the resulting XRD patterns before and after calcination for the 1, 5, and 10th cycles, and c. the FT-IR spectra. TEM images and inset high-resolution TEM of d. MgO calcined at 600 °C without milling, e. MgO regenerated at 600 °C after the 1st cycle, and f. MgO regenerated at 600 °C after the 10th cycle.

HR-TEM analysis was further conducted to determine the crystalline phase after the regeneration cycles. HR-TEM analysis for MgO before and after milling and regeneration cycles, as shown in Figure 4d-f, exhibits an interlayer spacing of 0.21 nm that corresponds to the (220) lattice plane of MgO (reference PDF card No. 00-045-0946).^50^ Therefore, the MgO milling and regeneration cycles induce only morphological change without altering the bulk crystalline phase of MgO, consistent with the XRD patterns shown in Figure 4b. A small decrease in capture capacity was observed for the last cycles, which can be explained by the presence of a small fraction of iron, originated from jar abrasion, that is inactive for adsorption, but slightly increases the mass in the material observed using EDX (Figure S5). While several articles have reported enhanced CO_2_ capture using trace amounts of ZrO_2_ due to an increase in the specific surface area, none provided clear evidence of Zr incorporation into the MgO framework. A mixture of MgO and ZrO_2_ was obtained with a small ZrO_2_ content.^51,52^ This aligns well with our results. Indeed, no zirconium was detected in the MgO crystals by EDX analysis, and no macroscopic ZrO_2_ particle was observed (Figure S5). Over many cycles, impurities (iron and possibly ZrO_2_) abraded from the equipment would dilute the active MgO sorbent and thus reduce the mass-normalized capacity. Thus, periodic separation of the contamination would be required for practical application, which should be possible due to the density difference between ZrO_2_ and iron on the one hand and MgO on the other.

This robustness in capture performances seems to overcome a common limitation of highly engineered “high surface area MgO”, where over repeated utilization rounds followed by regeneration, particles tend to sinter, loose specific surface areas, and therefore offer degenerated capture performances.

The robustness of the mechanochemical approach reported in this work holds promise of a more resilient material, even if the recycling study in this work only tested the material for 10 runs. Indeed, although no significant changes were detected in our results, the potential influence of iron species on CO_2_ uptake and cyclic stability, through either catalytic or inhibitory effects on carbonation or regeneration, cannot be completely excluded.

## Conclusion

In this study, the effect of ball-milling on the CO_2_ capture performance of commercial MgO was investigated. Milling MgO under a CO_2_ atmosphere resulted in a higher CO_2_ uptake than static exposure by the MgO after milling under inert conditions. As a comparison, the use of milling during CO_2_ sorption allows one to enhance capture capacity with a 6-fold increase, in comparison with the adsorption without any milling and without any modification of MgO. Variations in milling parameters, including ball size, ball material, and milling frequency, affect the amount of CO_2_ adsorbed. Systematic variation of milling parameters reveals that efficient mechanical energy transfer, governed by milling frequency, ball size, and ball material, is essential for maximizing the capture performance. However, the use of a too high frequency leads to abrasion of the jar and the balls, leading to impurities in the sorbent. Furthermore, the addition of water during milling has a strong influence on the capture performance, as 5-fold more CO_2_ is captured under milling when the gas contains moisture. This technology appears to be a key benefit for CO_2_ capture for moist flue gas streams. For a milling time of 8 h, a value as high as 295.3 mg of CO_2_ captured per gram of sorbent is achieved, which is the highest reported value in the literature using commercial MgO. Under optimized conditions, MgO exhibits good cyclic stability for conditions approaching those encountered in industrial flue gas streams, retaining nearly constant CO_2_ uptake over ten consecutive capture and regeneration cycles, with an average sorption capacity of 17.4 wt %. Structural analyses indicate that carbonation proceeds via the formation of magnesium carbonate, bicarbonate, and hydroxycarbonate species, which can be fully decomposed back to MgO upon calcination, while no structural damage of the sorbent is observed after 10 cycles. This highlights the practical relevance of the approach and its potential compatibility with point source CO_2_ capture. Numerous technologies are currently being explored for CO_2_ capture and sequestration, including absorption over amines and adsorption using porous materials, such as MOFs, activated carbons, or zeolites. Key criteria for sorbent selection include commercial maturity, ease of energy-efficient regeneration, accessible CO_2_ purity, customizability, environmental friendliness, sorbent stability, and affordability. While MgO meets nearly all of these requirements, making it highly competitive against other materials, its initial CO_2_ uptake is relatively low, which limits its large-scale application for CCS compared to MOFs or amines, for instance. However, mechanochemical processing to enhance its surface area significantly improves its performance, rendering it competitive with that of established porous materials for CO_2_ uptake. This method is certainly still far from practical applicability, and a number of additional factors still need to be explored. However, because of the promising results reported here, it seems to be an avenue worthy of further exploration.

## Supplementary Material



## Notes

Setup for experiments with moisture, analytical data from FT-IR spectra, and electron microscopy images (PDF)

## Notes

Dedicated to the memory of Edwin Chandross, who had a deep interest in many fields of science, including climate change and technologies to fight it.
