# Supplementary material for: Increasing the (Re)Activity of MgO for Carbon Capture under Continuous Ball Milling
Source: Chem Mater. 2026 Oct 8;38(20):10576–84. doi: 10.1021/acs.chemmater.6c01597 (PMC13718985; doi:10.1021/acs.chemmater.6c01597)
Supplement: Supplementary file 1 [file cm6c01597_si_001.pdf]

Increasing the (Re)Activity of MgO for Carbon Capture under Continuous Ball Milling

Dr. Emanuele Antico,[1‡] Dr. Thibaud Aumond,[1‡] Marc Meyer,[1] Dr. Linfeng Li,[1] Dr. Eko Budiyanto,[1] Prof. Ferdi Schüth[1]*

[*]schueth@kofo.mpg.de

[‡] These authors contributed equally and are displayed in alphabetical order.

1 Max-Planck-Institut für Kohlenforschung, Kaiser-Wilhelm-Platz 1, 45470 Mülheim an der Ruhr, Germany.

SUPPORTING INFORMATION

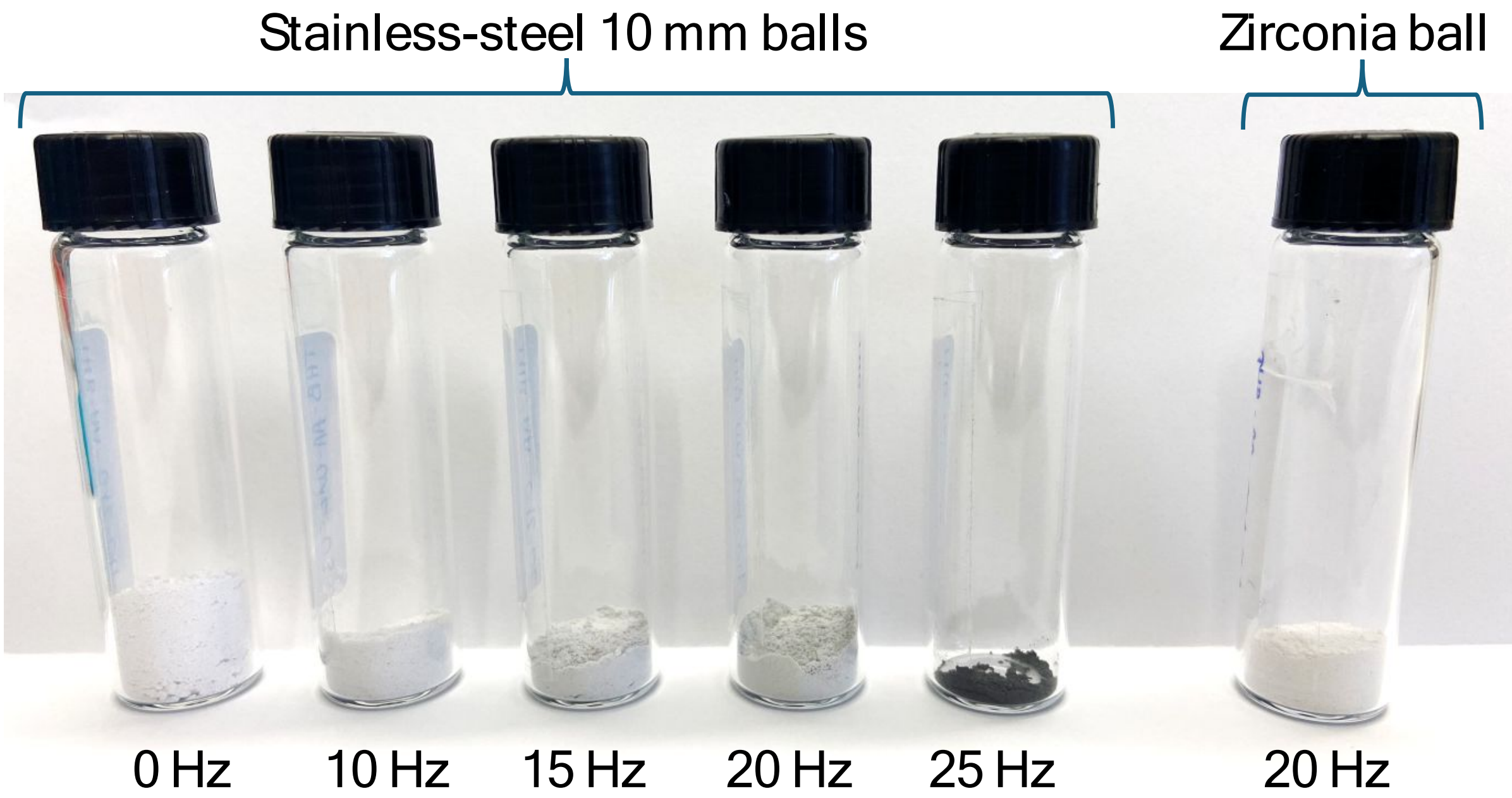

Figure S1. Photos of the sorbent after milling under different frequencies for 1 hour. In the right side, the resulting powder using the zirconia ball of 20 mm.

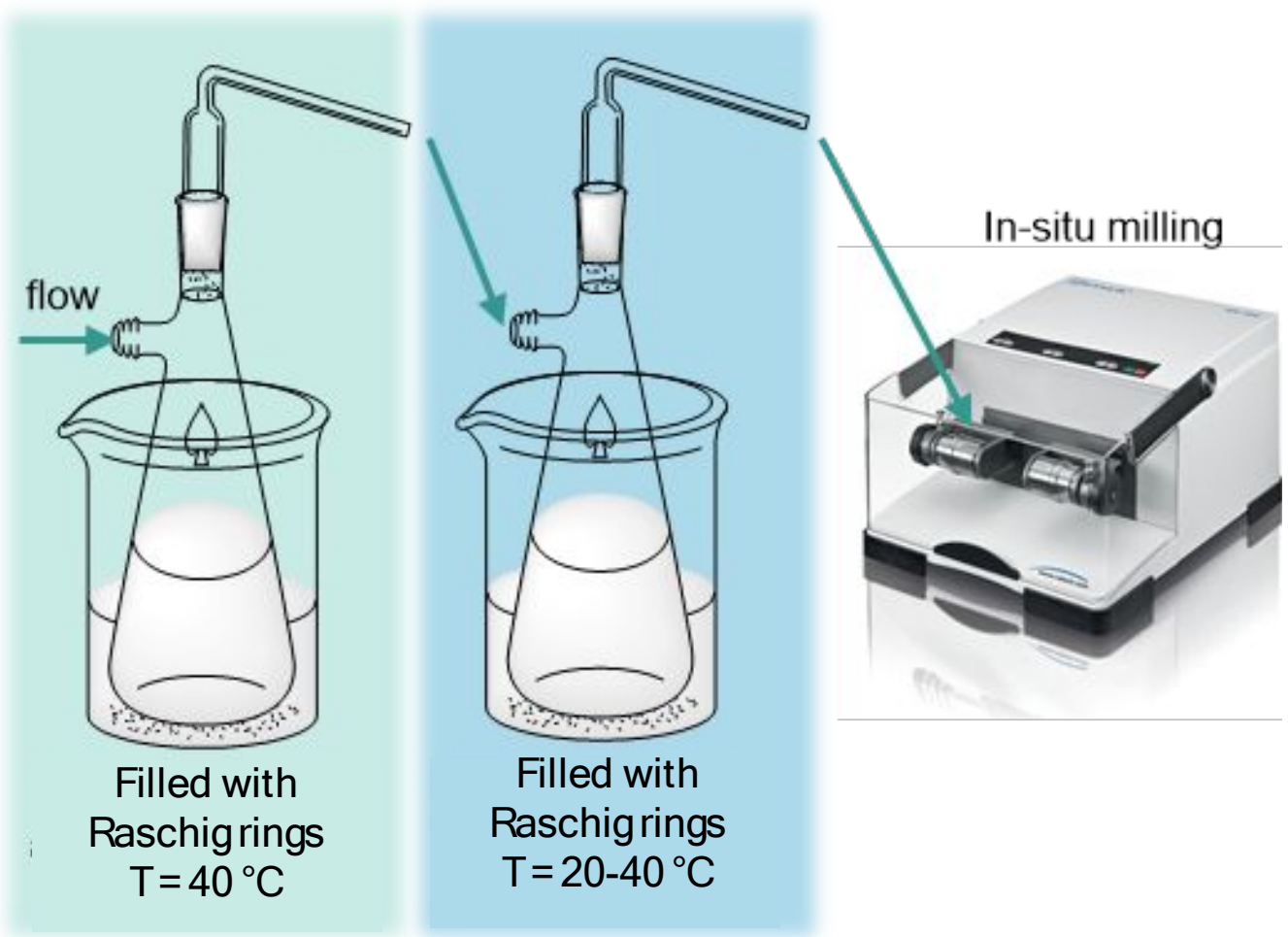

Figure S2. Scheme of the setup used to generate water in the gas flow. The temperature of the first bubbler was fixed to 40 °C while the temperature of the second was changed between 20 and 40 °C.

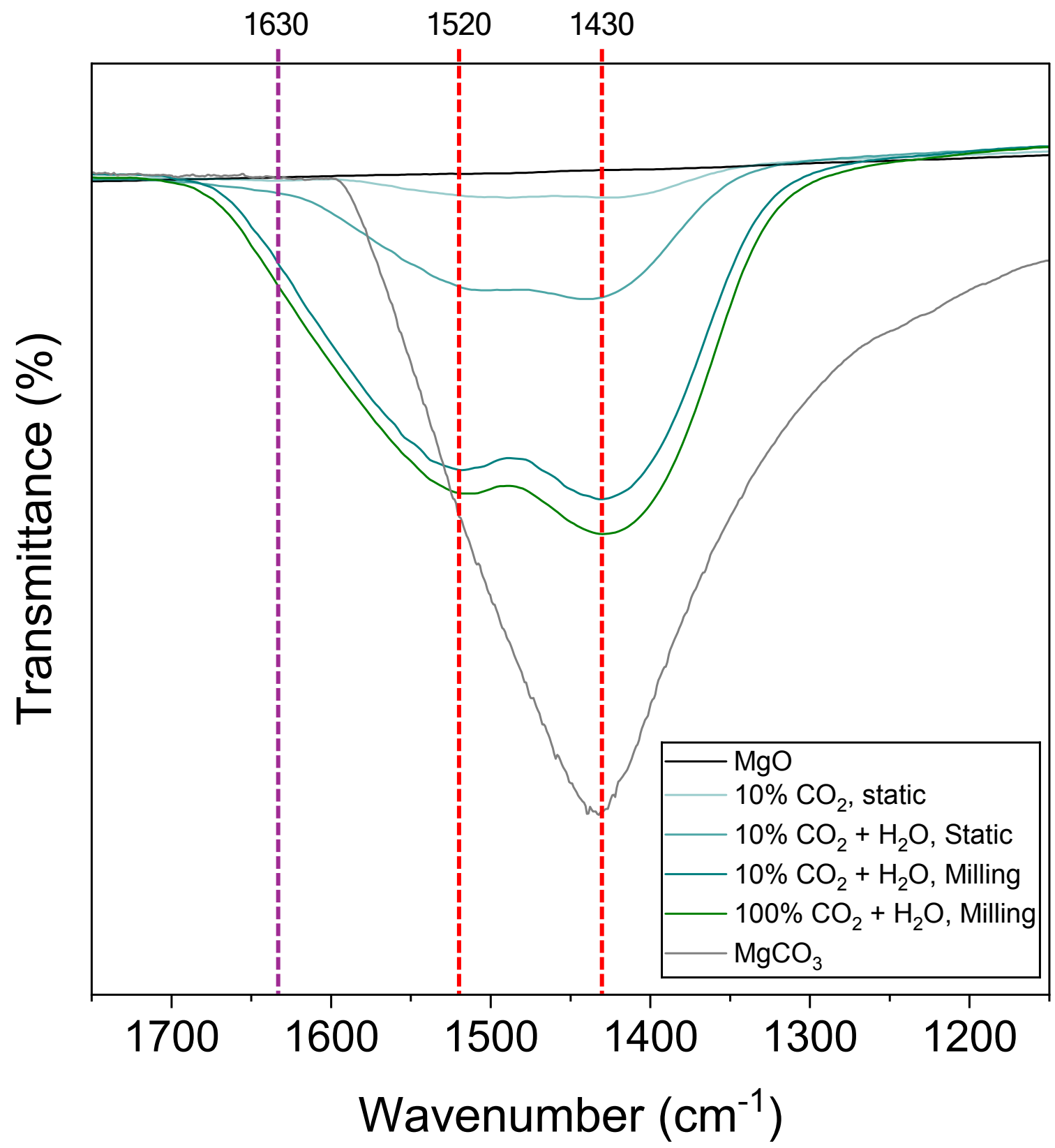

Figure S3. Zoom on the FT-IR spectra obtained after different capture parameters.

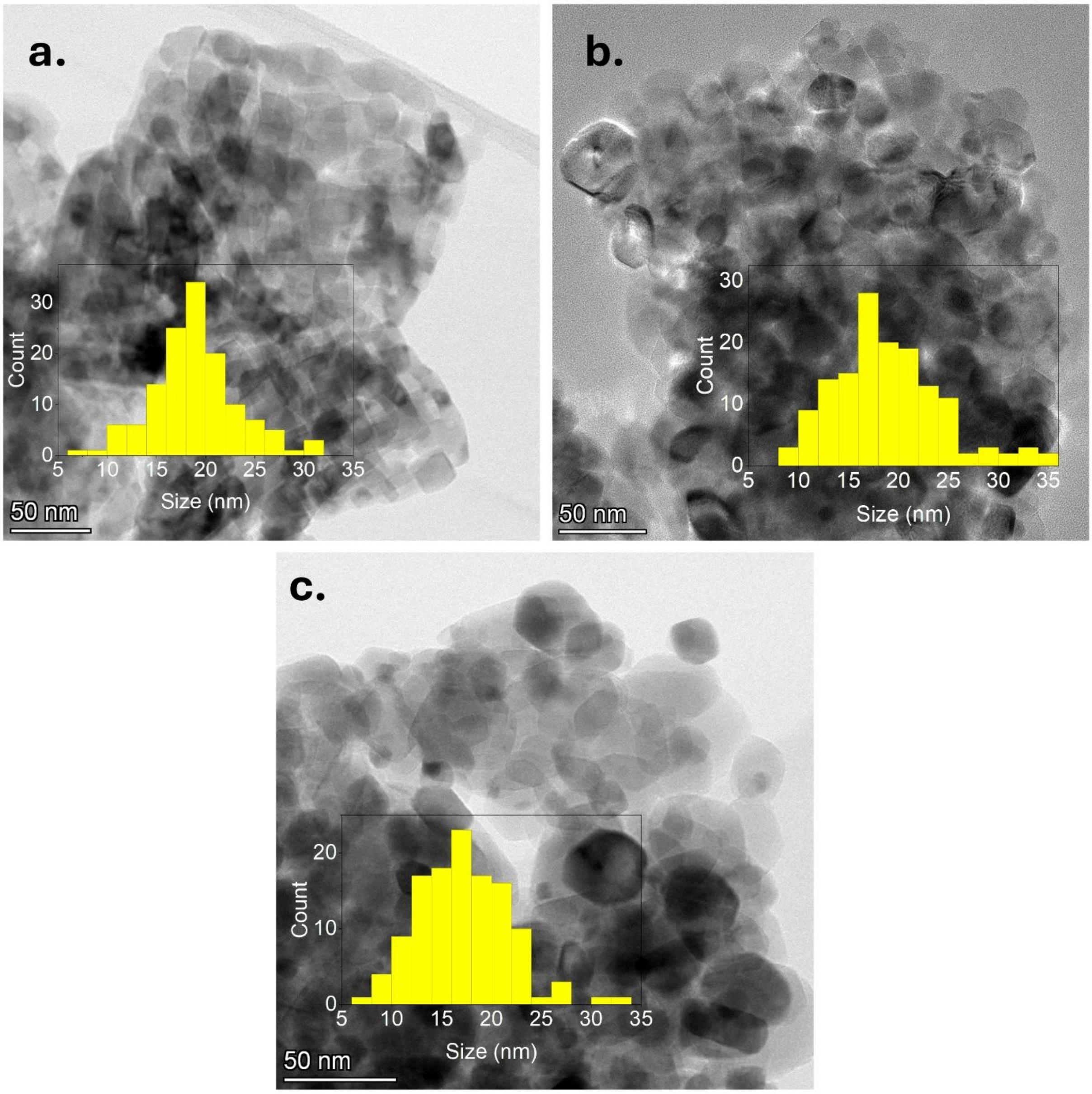

Figure S4. Low magnification TEM images and estimated size distribution of a. MgO calcined at 600 °C without milling, b. MgO regenerated at 600 °C after the 1st cycle, and c. MgO regenerated at 600 °C after the 10th cycle.

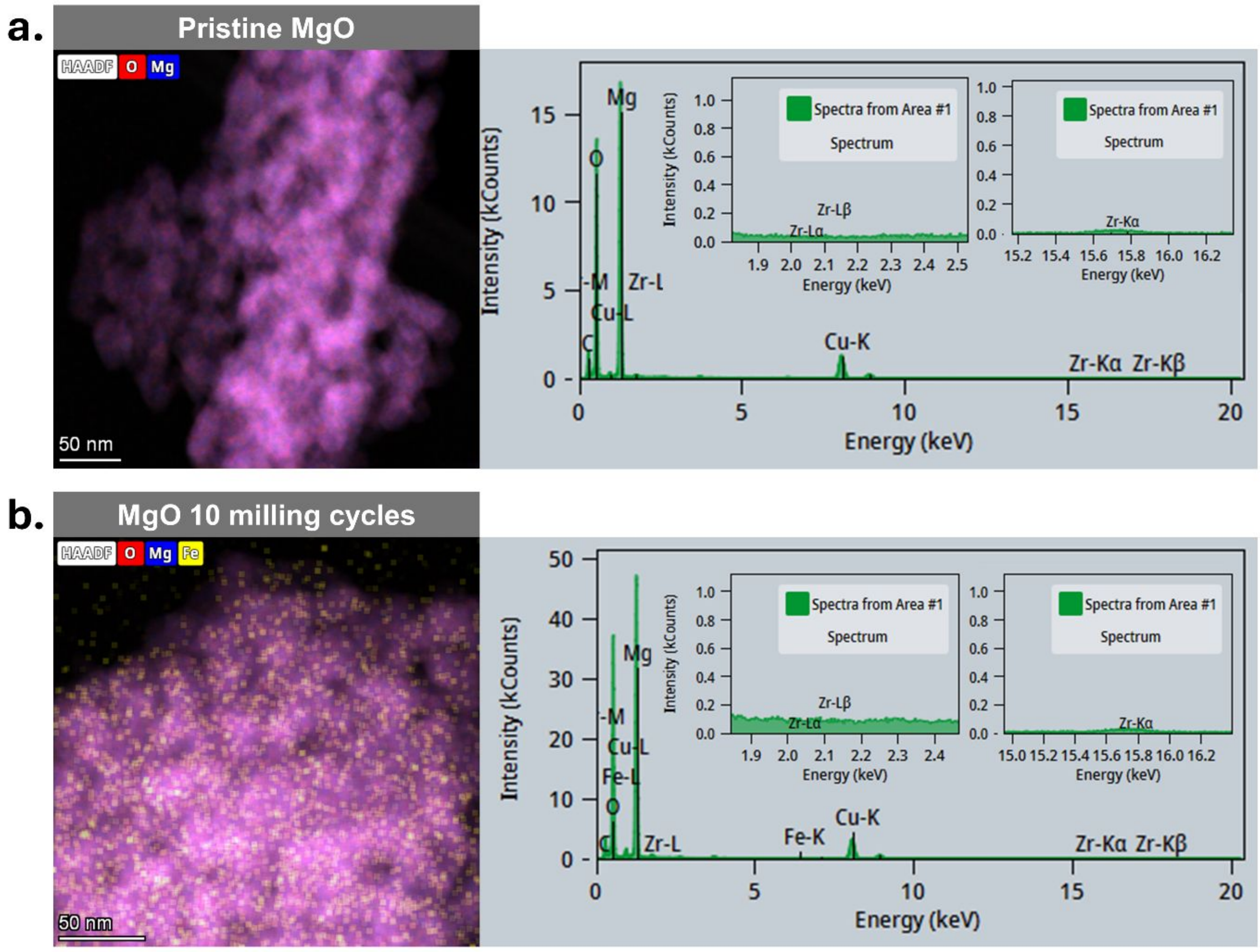

Figure S5. STEM–EDX elemental mapping with corresponding EDX spectra, including insets highlighting the Zr-L and Zr-K lines, for (a) pristine MgO and (b) MgO after 10 milling cycles followed by calcination at 600 °C. In both samples, no detectable Zr-L peak is observed, while a comparably weak Zr-K signal is present in both samples from the instrumental background.
